# One-Step Synthesis of TiO_2_/SiO_2_-np Nanocomposite Photocatalytic Multilayer Films: Effect of Incorporation Time Sequences of SiO_2_ Nanoparticles during the TiO_2_ Film Growth

**DOI:** 10.3390/ma17061227

**Published:** 2024-03-07

**Authors:** Shutong Lai, Eric Aubry, Olivier Sublemontier, Pascal Briois

**Affiliations:** 1 Institut FEMTO-ST, UMR 6174 CNRS, Université de Franche-Comté, UFC, 2 Place Lucien Tharradin, Site de Montbéliard, 25200 Montbéliard, France; eric.aubry-01@utbm.fr; 2 CEA Paris Saclay, CNRS, NIMBE, 91191 Gif-sur-Yvette CEDEX, France; olivier.sublemontier@cea.fr; 3 Institut FEMTO-ST, UMR 6174 CNRS, Université Technologie de Belfort Montbéliard, UTBM, Rue Ernest Thierry Mieg, Site de Montbéliard, 90010 Belfort CEDEX, France; pascal.briois@utbm.fr

**Keywords:** nanocomposite film, sputtering, TiO_2_, photocatalysis, aerodynamic lens, SiO_2_-np

## Abstract

In this article, the TiO_2_/SiO_2_-np nanocomposite multilayer films were synthesized in a single step by reactive magnetron sputtering combined with a nanoparticle aerosol jet. The SiO_2_ nanoparticles (SiO_2_-np) were introduced into a growing TiO_2_ thin film with different time sequences during deposition for a fixed duration. The SiO_2_-np acting as impurities are introduced into the TiO_2_ to willingly disturb its growth and to cause growth defects in order to increase the specific surface area of the photocatalytic film. In reason of the non-photoactive properties of the SiO_2_ nanoparticles, their introduction allows us to study only the effects induced on the film morphology, microstructure, and photocatalytic properties by their incorporation. The fractographies and topographies reveal strong changes in the morphologies depending on the time sequence of the nanoparticle introduction in the thin films. The introduction of SiO_2_-np from the beginning of the TiO_2_ film growth leads to the formation of high and large growth defects resulting in a highly diffusive surface. In addition, XRD analysis shows that the crystallite size tends to decrease as the composite film layer gets closer to the surface. Their photocatalytic performance is obtained by following the degradation of orange G dye under UV-visible irradiation. The photocatalytic performance is not only related to the specific surface area of the catalyst film, and the coverage of the photoactive phase on the surface, but also to the crystal quality of the photoactive phase. Furthermore, the samples exhibit good photostability, maintaining the same activity after four degradation cycles. In the specific case of TiO_2_/SiO_2_-np, it is demonstrated that the introduction of the nanoparticles only at the beginning of the film growth is more efficient than a continuous introduction. This result suggests that this original process allows the use of a relevant strategy for the nanoparticle introduction according to the required functionality.

## 1. Introduction

Photocatalysis has been extensively investigated over the past few decades due to its potential for a wide range of applications, including environmental remediation (water purification), water splitting for hydrogen production, and self-cleaning surfaces [1,2]. Among photocatalysts, TiO_2_ with anatase structure has received significant attention due to its excellent properties such as strong oxidizing power, biocompatibility, low cost, and high efficiency under UV light. Compared to TiO_2_ powder, the TiO_2_ thin film offers several benefits in photocatalytic applications due to its substrate flexibility, stability, and the potential for modification [3,4,5]. One key factor that strongly influences the photocatalytic activity of TiO_2_ thin films is its morphology which may have a significant impact on its real developed surface, roughness, and charge transfer properties. Since the photocatalytic reaction mostly occurs on the surface of the photocatalysts, the properties of the photocatalysts’ surface are the decisive factor for the photocatalytic performance [3,6,7]. Indeed, the active sites (adsorption or electronic charge transfer) are essential in the photocatalytic reaction. The enhancement of their performance is the subject of numerous research [8,9,10]. In addition, the number of active sites also limits severely the performance of the photocatalytic thin films.

Many deposition methods for synthesizing TiO_2_ thin films with different morphologies have also been studied by varying some parameters, such as, for sol-gel and CVD technique, the effect of the annealing temperature [11,12,13,14]; for spray pyrolysis technique, the film morphology has been evaluated by changing the synthesis temperature [15,16]; for PVD techniques, studies have been carried out on film morphology by changing the working pressure, oxygen partial pressure, sputtering time, and DC power [17,18,19]. The TiO_2_ film morphology can also be altered by doping [20,21,22] or by incorporating nanoparticles to create nanocomposite films [23,24,25,26,27,28]. However, both methods, usually not only change the morphological characteristics of the film, but also affect other properties of TiO_2_, i.e., optical [22,23,24,25], photocatalytic [20,21,22,23,24,25], and surface energy properties [26,27,28]. Indeed, the nanoparticles behaving as impurities or dust particles disturb the film growth. These nanoscale local defects propagate through the film thickness, leading to micron-sized nodular growth defects on the film surface, resulting in changes in the thin film morphology [29,30]. These multiple and interconnected changes make very complex the understanding of the mechanisms leading to the enhancement or the degradation of the thin film properties.

In view of separating the effect of the nanoparticle introduction on the morphology from other required features, the influence of the SiO_2_ nanoparticle introduction on the growth of sputtered TiO_2_ thin film is discussed in this article. The nanocomposite film synthesis process combines a PVD process with a divergent nanoparticle jet which allows easy control of the nanoparticle incorporation into a matrix film [31,32,33]. The moment of the nanoparticle introduction in the growing film for a constant duration has been changed. The SiO_2_-np has been selected for its non-contribution to photocatalysis (wide band gap, no light absorption, and chemically inert [34,35]), and thus will not affect the photocatalytic properties of the TiO_2_/SiO_2_-np films. The observation of the changes induced by the nanoparticle introduction on the morphology, and on the structural, optical, and photocatalytic properties suggests that a proper sequential introduction is more efficient than a continuous introduction of nanoparticles.

## 2. Materials and Methods

### 2.1. TiO_2_/SiO_2_-np Nanocomposite Thin Film Synthesis

**Figure 1:**
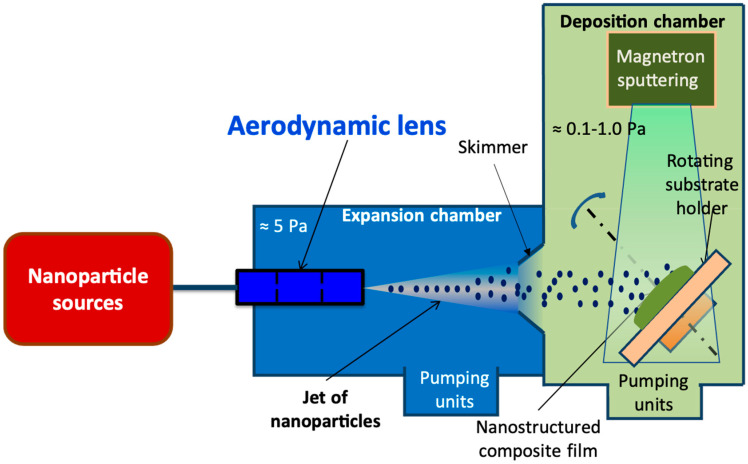
Drawing of the process for nanocomposite thin film synthesis allows independent control of nanoparticles and matrix deposition [31].

The principle of the process for nanocomposite film synthesis is shown in Figure 1. It combines an aerosol-based divergent nanoparticle jet with the magnetron sputtering technique. The nanoparticle source consists of an aerosol generator (AGK 2000, PALAS, Karlsruhe, Germany) alimented by a suspension of nanoparticles. The aerosol generator is connected to an expansion chamber maintained at 5 Pa with a multistage roots primary pump of 40 m^3^ h^−1^. Within the expansion chamber, a custom-made aerodynamic lens transports the nanoparticles to the substrate in the sputtering chamber [31,32,33].

The standard aerodynamic lens consists of several diaphragms with successively decreasing diameters through which the nanoparticles’ gas flow is increasingly concentrated on the central axis, forming collimated nanoparticle jets. A divergent nanoparticle jet is obtained by adjusting the diameter of the last diaphragm of a classical aerodynamic lens, which allows the large surface treatment [36,37] with homogenous deposition.

The magnetron cathodes placed in the deposition chamber are responsible for the synthesis of the matrix thin films. The combination of an aerodynamic lens and magnetron sputtering technique is possible because of their compatible working pressure and deposition rate. This process of nanocomposite thin film synthesis allows the independent control of nanoparticle and matrix deposition.

### 2.2. Substrate Preparation

A soda-lime glass substrate, measuring 75 × 25 mm^2^, was employed in the study. Before deposition, the substrate underwent a pretreatment involving the application of a 400 nm SiN_x_ layer. This SiN_x_ layer served as a barrier to prevent sodium diffusion [38,39,40].

### 2.3. TiO_2_ Film Synthesis

TiO_2_ matrix synthesis was conducted by pulsed DC reactive magnetron sputtering technique using 2 magnetron cathodes placed symmetrically in front of the movable substrate holder. The targets are in Ti (99% purity) with a dimension of 100 × 200 mm^2^ and powered with a pulsed DC Advanced Energy dual power supply.

A mix of high-purity oxygen and argon were used as the sputtering gas. The gas flow rates were controlled with MKS MF-1 flowmeters. Before deposition, a base pressure of 10^−4^ Pa was obtained with a turbomolecular pump. The base and sputtering pressure were measured with a wide range gauge (Edwards) and Baratron gauge, respectively.

The deposition time was fixed at 60 min. The main deposition conditions are summarized in Table 1. The increase in the substrate temperature is due to the energies dissipated by the atoms bombarded by the sputtering without adding an external heat source. A substrate temperature of about 300 °C is reached at the end of the deposition. To ensure the uniformity of the synthesized layer on a microscope glass slide, the substrate holder was laterally moved in front of the cathodes.

### 2.4. SiO_2_ Nanoparticle Incorporation

The SiO_2_-np suspension used in the experiment was prepared by mixing SiO_2_-np powder, commercially purchased from EVONIK Industries (Essen, Germany), with absolute ethanol (99%). The SiO_2_-np powder had an average size of 90 nm. The concentration of the resulting suspension was maintained at 1.0 g·L^−1^. Notably, there were no visible aggregates of SiO_2_-np observed in the colloidal suspension, indicating a well-dispersed state. The aerosol generated from the SiO_2_-np suspension forms a divergent SiO_2_-np jet by the aerodynamic lens toward the substrate in the sputtering chamber. More detail of the nanoparticle jet is given in the supplementary information.

### 2.5. TiO_2_/SiO_2_-np Nanocomposite Architecture

**Figure 2:**
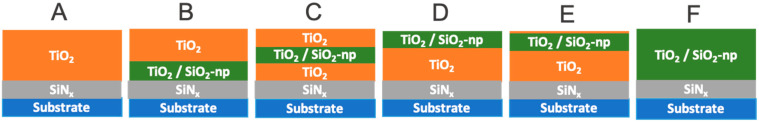
Illustration of prepared TiO_2_/SiO_2_-np nanocomposite samples having different architecture types.

As mentioned before, this process allows an easy management of the nanoparticle introduction in the growing film, so, different ways of nanoparticle incorporations have been realized. In this study, a sequential nanoparticle incorporation at different times during the TiO_2_ deposition is evaluated and compared to a continuous incorporation. The different architectures of nanocomposite film are illustrated in Figure 2. Layers of the same color in the coatings are made in the same way. The deposition time is fixed at 60 min. Sample A corresponds to the bare TiO_2_ film which can be considered as a reference. In contrast, sample F corresponds to a continuous incorporation of nanoparticles to form a TiO_2_/SiO_2_-np nanocomposite thin film. By using Energy Dispersive Spectroscopy (EDS), the concentration of SiO_2_-np in this coating (sample F) is approximately (to within a few at. %) estimated at 13.2 at. %, from measuring the Si concentration in the same nanocomposite film deposited on Fe substrates containing neither Si nor Ti. Samples B, C, and D are synthesized with a 20 min incorporation of SiO_2_-np at different times during the TiO_2_ deposition: sample B, with the incorporation of SiO_2_-np for the first 20 min of deposition, sample C with the incorporation of SiO_2_-np for the second period of 20 min of deposition, and sample D with the incorporation of SiO_2_-np for the last 20 min of deposition. Sample E is almost the same as sample D, the only difference is that sample E is finished by a thin layer of TiO_2_ (≈20 nm) on the surface.

### 2.6. Characterization of the Nanocomposite Films

The crystal structure of the films was characterized using X-ray diffraction (Bruker D8 Focus equipped with LynxEye detector (Bruker, Billerica, MA, USA), Co K_α1+α2_ radiation in θ−2θ configuration, 0.02° step). From the Full-Width at Half-Maxima (FWHM) of the diffraction line, the average crystallite size was also estimated considering spherical crystallites independent of crystallographic directions. The instrumental contribution on the line broadening was assumed negligible compared to that induced by the crystallite size. The dislocation density and the micro-strain are also calculated for a better understanding of the crystal quality. The top surface and cross-section micrographs of the prepared thin films were observed via Field Emission Scanning Electron Microscopy (Jeol JSM-7800 F, JEOL, Tokyo, Japan). The thickness of the samples was estimated from their brittle-fracture cross-section images. The total transmittance and the diffuse reflectance measurement of the samples are performed with the Ultraviolet-Visible-Near Infrared spectrophotometer (Shimadzu UV-3600, Shimadzu, Kyoto, Japan). Based on the diffuse reflectance measurement R, the bandgap Eg of the nanocomposite films is determined by using the Kubelka-Munk method (Equation (1)) which is particularly well suited for rough samples. *F*(*R*) corresponds to the Kubelka-Munk function [41,42]. The bandgap energy *E_g_* in eV is calculated from the Equation (2), where *h* is the Planck constant (6.63 × 10^−34^ m^2^ kg s^−1^), ν is the light frequency (Hz), and *A* is a constant:

(1) $$F(R)=\frac{{\left(1-R\right)}^{2}}{2R}$$

(2) $${\;(\alpha h\nu )}^{\gamma }=A\;(h\nu -E_{g})$$

For direct transition, γ is equal to 2 and 0.5 for indirect transition. By plotting ${\left(\alpha h\nu \right)}^{\gamma }$ as a function of the photon energy hν, the bandgap value can be determined from the extrapolation of the linear line portion of the curve to zero absorption coefficient. To estimate the roughness and the developed surface of the thin films, the profilometry (Altisurf 500, Altimet, Santiago, Chile) is also used. For all samples, the scanning surface was 200 × 200 µm^2^ using a microforce probe, with a step size of 500 nm which was the shortest step size that could be used. Because the minimum step size limits the accuracy of the measurement, it only gives an approximate roughness and developed surface result. A software connected with the profilometer was used for data processing and generated a 3D image from which the average value of Sq (root mean square height) and the developed surface were calculated. The Sq value corresponds to the standard deviation of the height and can be used as an indication of the surface roughness. Atomic force microscopy has been implemented without success, mainly due to the fact that the size and roughness of the protrusions are beyond the accurate range of AFM measurements.

### 2.7. Photocatalytic Tests

The photocatalytic performance of the samples is estimated by following the degradation of Orange G dye in contact with a photocatalytic film under UV-visible irradiation (Xenon lamp, 150 W) for 1 h at 30 °C. The stabilized light source from Quantum Design (Diego, CA, USA) produced a constant spectral irradiance from 300 to 800 nm over the time of the experiment. The distance between the film surface and the irradiation source is approximately 10 cm, and the intensity of light at the film surface is 20 kW/m^2^. This dye was chosen for its stability and difficulty to degrade. It is widely used in the printing and textile industries [43]. The photolysis test performed with a bare glass substrate shows that the dye is stable under irradiation and that the dye degradation is only induced by the photocatalytic process. The absorbance of Orange G dye is recorded every 18 s at 485 nm which represents the maximal absorbance wavelength of the Orange G dye. According to the Beer-Lambert equation, the dye concentration C is linearly dependent on the absorbance value A (Equation (3)). The Orange G dye volume is about 70 mL with a concentration of 20 mg L^−1^ with a natural pH of 5.6. The sample is immersed in the dye solution for 30 min in the dark before the photocatalytic test to ensure the establishment of an adsorption/desorption equilibrium. According to the experimental conditions, it is considered that the dye adsorption is limited on the TiO_2_ surface. The degradation reaction proceeds with intermediate species such as hydroxyl radicals. In our specific photocatalytic reactor case, photocatalytic dye degradation follows pseudo-first-order kinetics. The Langmuir–Hinshelwood kinetic model [44] is used to calculate the photocatalytic performance (Equation (4)). The apparent rate constant *k_app_* indicates the chemical reaction rate and represents the photocatalytic performance. The stability of the photocatalysts was tested by repeating 4 times the same experiment once each run of the photodegradation experiment finished.

(3) A=εlC

where *ε* is the molar absorption coefficient (m^2^ mol^−1^), *l* is the path length of the cell (m).

(4) $$\ln\left(\frac{C_{0}}{C_{i}}\right)=kKC=k_{app}*t$$

where *C*_0_ and *C_i_* are the initial dye concentration and the dye concentration at time *i*, *t* is the time (min), K is the adsorption coefficient of the dye, *k* is the reaction rate, and *K_app_* is the apparent rate constant (min^−1^).

## 3. Results and Discussion

### 3.1. Structural Properties of the TiO_2_/SiO_2_-np Nanocomposite Films

**Figure 3:**
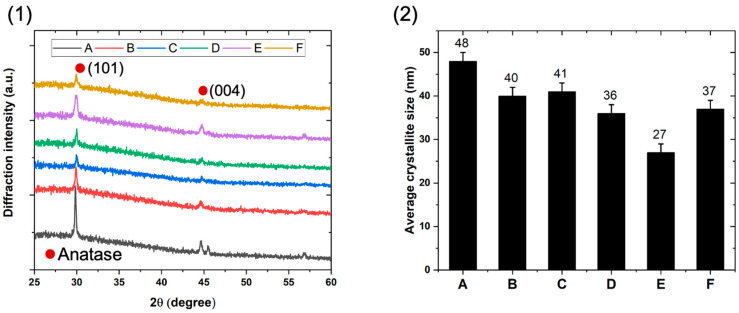
Structural properties of the nanocomposite TiO_2_/SiO_2_-np film: (**1**) X-ray diffractograms with θ−2θ mode; (**2**) average crystallite size of the TiO_2_ matrix.

The as-deposited thin films are all crystallized in the anatase phase (space group I4_1_/amd) which is consistent with the substrate temperature reached at the end of the deposition (~300 °C). No diffraction peaks corresponding to rutile or brookite phases are detected. The X-ray diffractograms shown in Figure 3(1) reveal that all the films crystallize with a preferential orientation along the (101) direction. The diffraction line intensities for nanocomposite films are lower than that of the TiO_2_ film. It is assumed that the growth disruptions produced by the incorporation of SiO_2_ nanoparticles would lead to a poorer crystallization of the nanocomposite films due to a more disordered crystalline and higher defective structure). Since the SiO_2_ nanoparticles are amorphous, the observation of the SiO_2_ is not possible. The average crystallite size of the anatase photoactive phase is determined using the Scherrer formula (Equation (5)) from the FWHM of the (101) diffraction line [11], considering spherical crystallites with homogeneous microstructure independent of the crystallographic directions:

(5) $$D=\frac{K\lambda }{\beta \;cos\theta }$$

where *D* is the average crystallite size, *K* is the Scherrer constant, *λ* is the X-ray wavelength (nm), *β* is the FWHM of diffraction line intensity, and *θ* is the Bragg angle of the considered diffraction line. As shown in Figure 3(2), the different architectures have an impact on the crystallite size. First of all, sample A has the largest grain size (48 ± 2 nm), whereas the nanocomposite film carried out with a continuous flux of SiO_2_ nanoparticles (sample F) and samples D and E (nanoparticles introduced in the last 20 min of deposition) exhibit the smallest crystallite sizes (27 to 37 ± 2 nm). Furthermore, the average crystallite size of the nanocomposite films finished with only TiO_2_ (B and C) are close and higher than that of samples D, E, and F. These results demonstrate that the incorporation of nanoparticles of tens of nanometers limits slightly the TiO_2_ matrix crystallite growth. The crystallite size of sample E is smaller than that of sample D, probably due to the very fine TiO_2_ film on the extreme surface which should have a very fine crystallite size.

The micro-strain (σ) developed in the thin films could be estimated from Equation (6) [11], where *β* is the full-width at half-maximum of the (101) diffraction line and *θ* is the Bragg’s angle. The origin of the micro-strain is related to the lattice misfit.

(6) $$\sigma =\frac{\beta }{4\;tan(\theta )}$$

The misfit within the lattice of the crystal resulted in the formation of dislocation. The dislocation density (δ), defined as the length of dislocation lines per unit volume, represents the imperfection in a crystal. It can be approached by using Equation (7) [11], where *D* is the average crystallite size, and *a* is the factor that is equal to unity for minimum dislocation density.

(7) $$\delta =\frac{a}{D^{2}}$$

The calculated micro-stain and dislocation density values are shown in Table 2. For both micro-strain and dislocation density, they are inversely proportional to the average crystallite size. That means the defect concentration in the lattice increases with a decrease in average crystallite size. With respect to these results, sample A would have the lowest defect concentration. For other samples, due to the disturbance of the TiO_2_ crystal growth by the SiO_2_ nanoparticles, the defect concentration is more or less increased. Sample E would have the highest defect concentration in the lattice probably due to the very thin layer formed on the extreme surface of sample E.

### 3.2. Morphological Features of the TiO_2_/SiO_2_-np Nanocomposite Films

**Figure 4:**
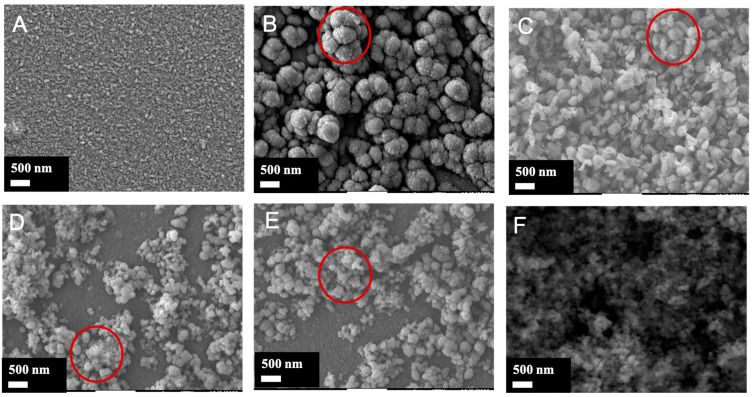
Top surface micrographs of the TiO_2_/SiO_2_-np nanocomposite films.

**Figure 5:**
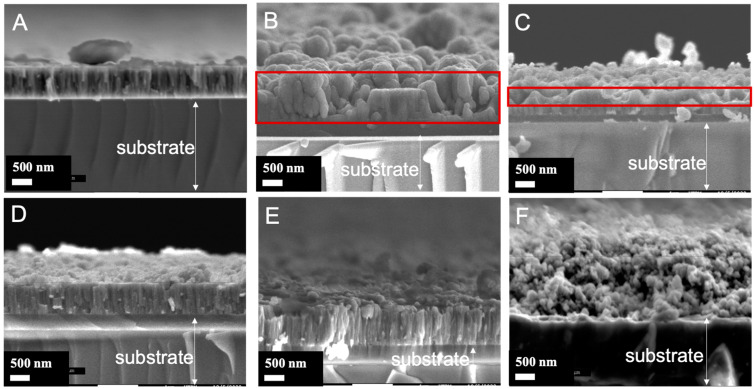
Brittle-fracture cross-section micrographs of the TiO_2_/SiO_2_-np nanocomposite films.

The top surface and cross-section micrographs of the nanocomposite films with different architectures are presented in Figure 4 and Figure 5, respectively. The bare TiO_2_ film (sample A) appears as a relatively dense thin film with uniform columnar grains, resulting in a faceted surface consistent with the deposition conditions (300 °C, 0.5 Pa). The morphological features correspond to the typical zone 2 of the well-known structural zone diagram [45]. The introduction of nanoparticles in the matrix film changes considerably this morphology regardless of the moment of the nanoparticle incorporation. For all nanocomposite films, more or less large protrusions on their surface are distinguishable. Figure 5 shows that the protrusions start at the interface film/substrate for film B, in film C around the middle of the coating, and only in the proximity of the surface for the D and E films. As for film F, due to the continuous incorporation, the entire film thickness is affected. The protrusions start to grow when the nanoparticles are introduced into the film, as expected. Then, it can be concluded that the earlier the protrusion growth starts, the larger the protrusion at the surface (film B). The D and E films grown with the introduction of nanoparticles for the last 20-min period exhibit smaller protrusions, some parts of the surface remaining unaffected. When a continuous introduction of nanoparticles is used, the morphology gets closer to a nodular morphology. The columnar grains are no longer observable, and the morphology consists of nodules of about 30 nm diameter. The probability that a nanoparticle condenses in the vicinity of the growth defect zone becomes high, limiting the constant growth of the matrix film and resulting in a sponge-like or nodular morphology.

**Figure 6:**
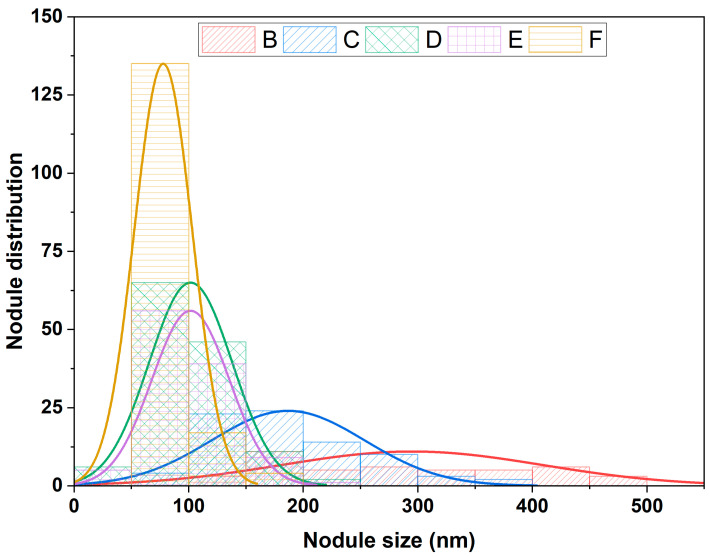
Protrusion size distribution of TiO_2_/SiO_2_-np nanocomposite films.

**Figure 7:**
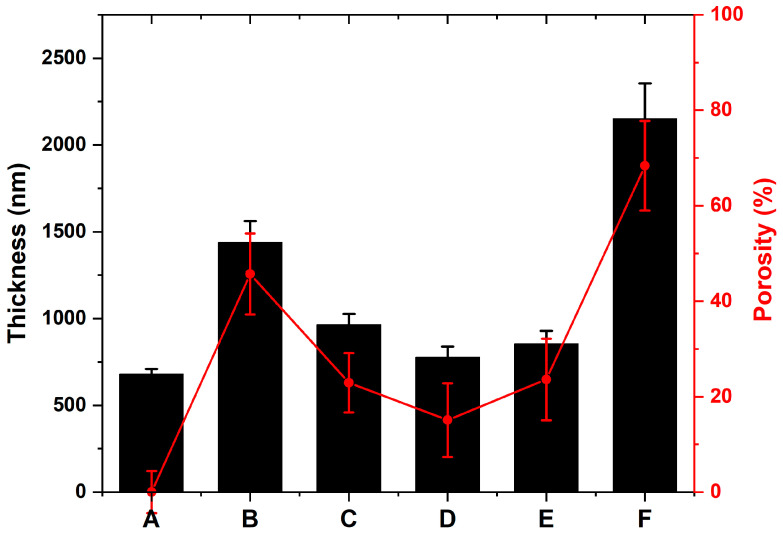
Film thickness and related porosity of TiO_2_/SiO_2_-np nanocomposite films.

Based on the SEM image, the features of the protrusions were estimated using the image analysis software ImageJ (version 1.53 u) to define all protrusions on the sample surface. (cf. Figure 6). In film F, the average diameter of protrusions is the smallest and the number is the highest (strongly disturbed by the continuous nanoparticles flux). Logically, the protrusion features in films D and E are close. It can be observed that the earlier the nanoparticles are introduced, the larger the protrusions and their size range, however, their number decreases (films B and C). The protrusion width at the surface is proportional to its height (distance from the surface to the top of the nanoparticle seed). According to the basic model [46], the diameter of the nodular growth defect at the surface (W in nm) would be related to the size of the seed (d in nm) and the film thickness (t in nm) (cf. Equation (8)). A is a constant depending on the aspect ratio. Assuming homogeneous deposition on spherical seeds (A = 8), with a seed size of 90 nm (SiO_2_-np size), and the film thickness (Figure 7). The nodular growth defect diameter would be around 536 nm for sample B, around 379 nm for sample C, and 120 nm for sample D. This gives a rough approximation of the expected lateral size and is in general agreement with what is observed in the SEM images. The actual nodule size values are somewhat smaller than the calculated values, this may be due to the fact that the basic model theory is more applicable to a single growth defect. whereas in this case, the growth of the protrusion can be disturbed by other growth defects. Measurement errors can also contribute to the differences.

(8) $$W=\sqrt{Adt}$$

While the deposition time was the same for all coatings, the cross-section micrographs reveal strong disparities in film thicknesses. Figure 7 presents the evolution of the nanocomposite film thickness synthesized according to different architectures. The thickness of the pure TiO_2_ film is about 680 nm. The more the growth defects are large and high, i.e., the earlier are introduced the nanoparticles, the more the thickness increases to reach twice higher than that of the bare TiO_2_ film. Furthermore, the thickness of the F film is triple that of the bare TiO_2_ thin films. It is believed that only the height of the protrusion cannot explain such variation in thickness. Considering a constant sputtered particle flux condensing on the substrate (fixed time deposition, sputtering power, and substrate position according to the targets and nanoparticle flux), the porosity induced by the protrusion formation could contribute to this behavior. Comparing the thickness of the nanocomposite film with the bare TiO_2_ film allows for an approximation of the volume porosity that could be estimated according to Equation (9) (Figure 7). The volume porosity increases with the height and the width of the protrusion. Furthermore, the evolution of the diffraction line intensity of the nanocomposite film (see Figure 3) could be ascribed to the porosity in the film.

(9) $$Porosity=\frac{{Thickness}_{sample\;x}-{thickness}_{sample\;A}}{{Thickness}_{sample\;x}}$$

### 3.3. Optical Properties of the TiO_2_/SiO_2_-np Nanocomposite Films

**Figure 8:**
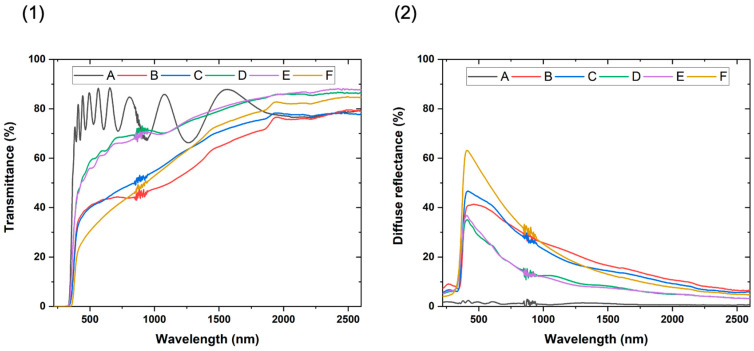
Optical properties of the TiO_2_/SiO_2_-np nanocomposite film: (**1**) the total transmittance; (**2**) the diffuse reflectance.

The optical properties such as the transmittance or the diffuse reflectance of the prepared samples are investigated by using a UV-VIS-NIR spectrophotometer. Figure 8(1) shows their total transmittance. The bare TiO_2_ film (sample A) exhibits a typical transmittance curve for a dense dielectric thin film deposited on glass with a SiN_x_ layer. The interference fringes of the transmittance are due to the partial light reflection/transmission at the film and substrate surfaces. The refractive index respects the classical dispersion laws. The relationship between the film thickness (*t*) and the number of fringes (*M*) and refractive index (*n*) is described in Equation (10) [47].

(10) $$t=\frac{M\lambda_{1}\lambda_{2}}{2(n\left(\lambda_{1}\right)\lambda_{2}-n\left(\lambda_{2}\right)\lambda_{1})}$$

With the introduction of the nanoparticles, the transmittance deteriorates especially as they are introduced from the initial stages of the matrix growth or if the nanoparticle insertion is continuous. The transmittance deterioration occurs mainly in the visible-near infrared range (400–2000 nm). This behavior is mainly ascribed to the surface and bulk light scattering by the numerous interfaces generated by the introduction of the nanoparticles and the growth defects. Furthermore, the refractive index would drop due to the mixture of the TiO_2_ film with the SiO_2_ nanoparticles or the pores having lower refractive indices. The well-known effective medium theories predicting effective refractive index in heterogeneous media would not be valid because of the non-negligible light scattering by pores and the too-high pore density [48]. The scattering occurs at the interface between two regions with differing refractive indices (pores or second phase such as nanoparticles) [49]. The effect of light scattering on the film transmittance is dependent on the particle size compared to the incident light wavelength. Three behaviors are distinguishable: (i) for particle size lower than the wavelength, the well-known Rayleigh scattering occurs; (ii) for particle size higher than the wavelength, light diffraction prevails; and (iii) for particle size of one order of magnitude of the wavelength, the van de Hulst approximation derived from the Mie’s theory would be applied [50,51,52]. According to the size of the protrusion, the light scattering would arise from a large range of defects. The evolution of the transmittance deterioration is consistent with the observed changes in morphology and porosity. The disappearance of the fringes, observable for the highest porous samples (B, C, and F), would then be caused by the light interference with the multiple pores inside the films. The observation of the slight fringes in samples D and F demonstrate that the morphology changes at the surface contribute partially to the transmittance deterioration compared to the bulk scattering.

Figure 8(2) shows the diffuse reflectance of the nanocomposite films, which are more sensitive to surface scattering. As expected, the nanocomposite films exhibit large diffuse reflectance. The results of the diffuse reflectance are consistent with the observation of the SEM images. Such as, the nodular or sponge-like morphology of sample F resulting from a continuous insertion of nanoparticles would have the roughest surface. The B and C samples consisted of large protrusions that exhibit a slighter diffuse reflectance meaning a lower surface roughness. However, the light diffusion in the infrared becomes the highest, probably due to the presence of large growth defects (see Figure 6). The surfaces of the D and E samples are less disturbed with the presence of clear parts, the diffuse reflectance is then lower. While these results are only qualitative, they allow the comparison of the developed surface which is an important parameter for photocatalysis.

### 3.4. Surface Feature Estimations

**Figure 9:**
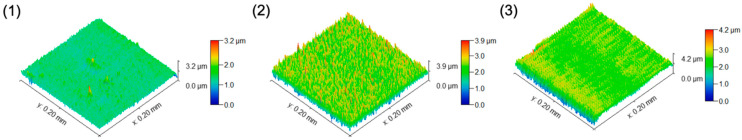
Selective 3D image (200 × 200 µm^2^) of the nanocomposite film surface obtained by profilometry: (**1**) sample A; (**2**) sample B; (**3**) sample F.

In order to further investigate the surface properties of the nanocomposite films, the surface roughness and the developed surface of the prepared samples have been estimated by profilometry, and the values were analyzed by the software that was integrated with the profilometer. Figure 9 shows the 3D images of selective samples A, B, and F treated by a modular program Gwyddion [53]. The 3D image of sample A is the smoothest of the three samples, while sample B is formed by dense narrow pinning (the protrusions), showing a rough and uneven surface. Sample F also exhibits a rough surface, but its surface seems more homogeneous than that of sample B. The accuracy of the profilometer does not allow the distinction of the nodules.

**Figure 10:**
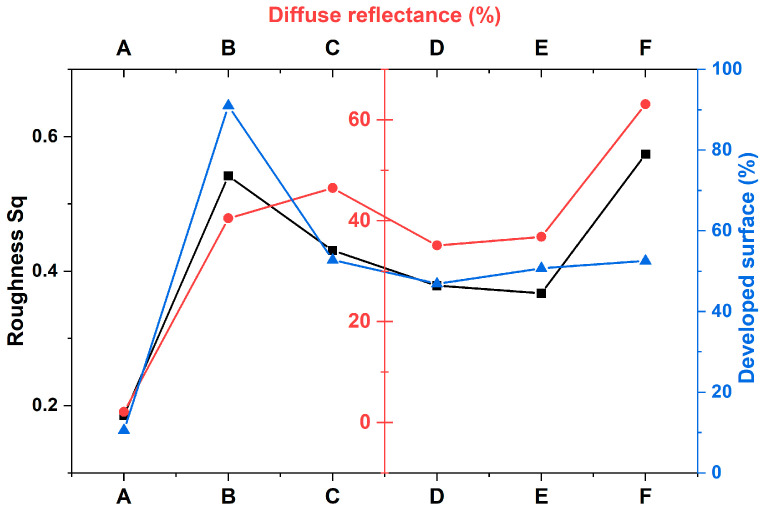
Correlation between the diffuse reflectance (at 410 nm) with the surface roughness Sq and the developed surface of the nanocomposites TiO_2_/SiO_2_-np films.

Figure 10 illustrates the evolution of the roughness (Sq) and the developed surface, along with their correlation with the diffuse reflectance. As mentioned previously, the diffuse reflectance of the nanocomposite films is sensitive to surface scattering. Despite the limited step size, it can be observed that the surface roughness (Sq) well increases with the size of the growth defect, as the film thickness evolves. The roughness difference between samples is mostly a result of the layer configurations. Additionally, the roughness correlates quite well with the diffuse reflectance, except for a slight deviation for sample B. At this point, it seems reasonable to believe that the diffuse reflectance qualitatively reveals the film surface roughness. The developed surface of sample B is nearly double that of sample F, while the roughness of sample F is slightly better than that of sample B. This well explains why sample F seems smoother than sample B from their 3D images. However, as the SEM image of sample F shows, sample F is composed of many small protrusions, which may exceed the accuracy of the contour measurement and lead to inaccurate measurement. It is then necessary to take the measurement results of sample F with a critical perspective.

### 3.5. Bandgap Calculations

**Figure 11:**
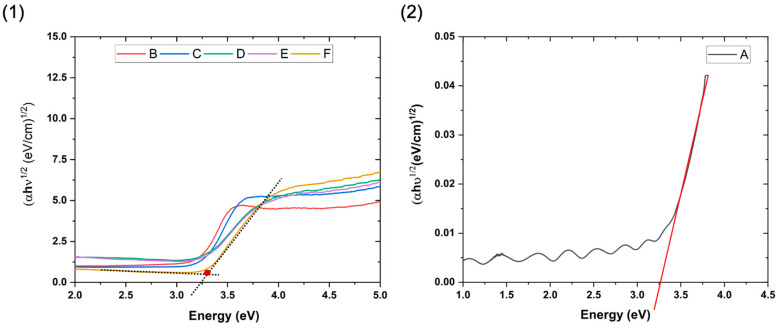
Tauc plot for band gap calculation (**1**) Kubelka-Munk method for porous nanocomposite thin films (**2**) standard method for smooth thin films.

The bandgap value of the film is estimated by the Kubelka-Munk function which is relatively well suited for powder or porous and rough or thin film samples. Assuming indirect transition for the anatase TiO_2_, ${\left(\alpha h\nu \right)}^{\gamma }$ = F (hν) has been plotted as shown in Figure 11(1). The *x*-axis intersection points of the linear fit of the Tauc plot give an estimate of the band gap energy. However, as introduced in [54], for a film constituted by 2 components, another method is more accurate to estimate the band gap value shown in Figure 11(1). In addition to a linear fit of the Tauc plot, a linear fit as a marker was also used for the slope below the fundamental absorption. The *x*-axis value of the intersection of the two fitting lines gives the band gap energy estimation. So, the band gap value is obtained by this method instead of a simple extrapolation described by the Tauc method. The bandgap of the bare TiO_2_ film (Figure 11(2)) is calculated from the total reflectance and transmittance using Equation (11), where t represents film thickness (cm). The bandgap values are shown in Table 3. The TiO_2_ bandgap value is 3.26 eV which is comparable with the theoretical band gap value which is 3.20–3.30 eV for the anatase TiO_2_ thin films [55,56]. For the nanocomposite films, the variation of the band gap values is almost negligible taking into consideration the measurement error. The introduction of SiO_2_-np has no significant effect on the optical bandgap values of the films. This may be due to the absence of close contact between SiO_2_-np and TiO_2_.

(11) $$\alpha \left(\lambda \right)=\frac{1}{t}\;ln(\frac{1-R\left(\lambda \right)}{T\left(\lambda \right)})$$

Furthermore, the well-known Urbach energy has been estimated by plotting $\ln\left(\alpha \right)$ = F (hν), shown in Table 3 as well. This shows the width of the band associated with the disorder. The values are consistent with values reported previously for sputtered delafossite thin films deposited at 380 °C [57] or in TiO_2_ [58]. It is shown that the disorder values increase when the nanoparticles are introduced in proximity to the surface, in relative agreement with the micro-strain and dislocation evolutions. Surprisingly, the disorder value is the lowest for the continuous introduction of the nanoparticles.

### 3.6. Photocatalytic Performance and Photostability

**Figure 12:**
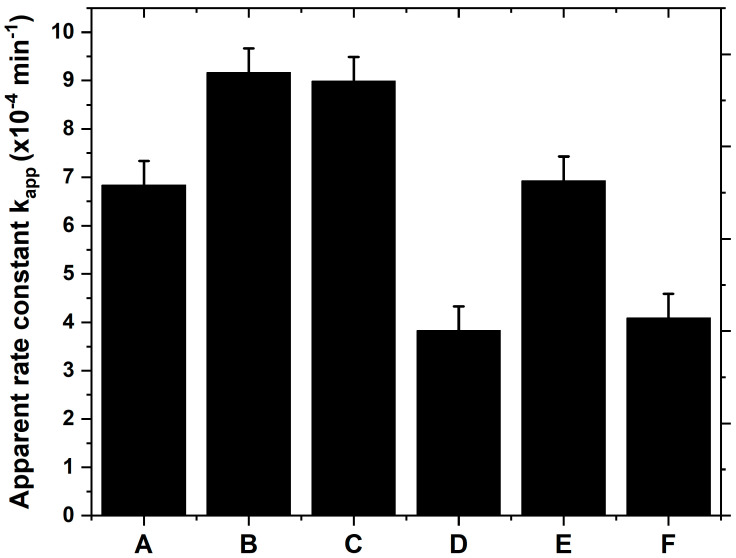
The apparent rate constant k_app_ estimated from the degradation of Orange G dye for the nanocomposite TiO_2_/SiO_2_-np samples.

The photocatalytic performance has been evaluated by following the degradation of the Orange G dye. This dye has been chosen for its stability to ultraviolet and visible light irradiation and its resistance [59]. The photocatalytic activity respects the Langmuir–Hinshelwood kinetic model, which is expressed by the apparent rate constant K_app_. Figure 12 plotted the measured K_app_ for the nanocomposite TiO_2_/SiO_2_-np films.

The ranking of photocatalytic performance from largest to smallest is as follows: sample B ≈ sample C > sample A ≈ sample E > sample F ≈ sample D. All samples with the mixture of TiO_2_ and SiO_2_ at the surface, i.e., samples D and F, exhibit poor photocatalytic activity. The presence of TiO_2_ at the surface, even for a flat surface such as for sample A, is thus important. The presence of the SiO_2_ non-photoactive phase reduces the coverage by the photoactive TiO_2_ phase and also acts on its microstructure by slightly decreasing the crystallite size and increasing the microstructural defect quantity. Indeed, the concentration of SiO_2_ is 13.2 at. %. This is consistent with the lower activity of sample D compared to sample E which exhibits similar surface properties. Furthermore, the increase of the surface roughness or the developed surface leads to the improvement of the photocatalytic activity of the films in which the surface is only constituted by the TiO_2_ phase. That is why, the K_app_ of sample B and sample C compared to that of sample A and sample E are higher. However, sample E shows a photocatalytic performance as sample A while sample E has a better surface roughness. This underlines the fact that another factor has to be into account, in addition to the developed surface and the TiO_2_ coverage. Indeed, the crystalline quality of the photoactive phase (crystalline defects, stress) may also help the enhancement of the photoactivity [60,61]. Table 3 implies that the crystal quality of sample E could be poorer than that of sample A by the presence of more dislocations and micro-strains.

Furthermore, another point should be considered, namely the wettability of the nanostructure. Indeed, the increase of the developed surface is carried out by generating growth defects having variable aspect ratios [62]. However, the changes in the aspect ratio would also affect the surface wettability. As a consequence, the contact surface with the dye could not exactly be as high as expected. This is a well-known problem for example in the microelectronic industry.

To sum up this part, several elements that determine photocatalytic activity are highlighted, reflecting the fact that photocatalysis is largely determined by the surface of the photoactive phase and its quality.

**Figure 13:**
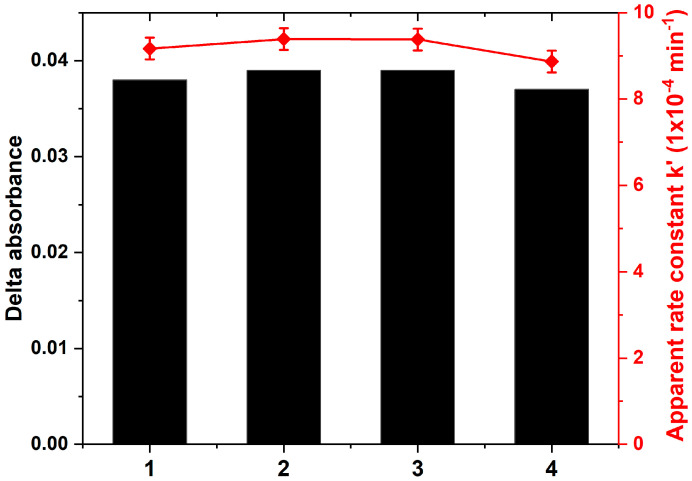
Photostability of the nanocomposite TiO_2_/SiO_2_-np film (sample B) of four cycles photocatalytic degradation.

The photostability of the samples was evaluated by conducting multiple photocatalytic cycles, and sample B was selected for this purpose. Figure 13 illustrates the results, showing that after four cycles, there was no significant decrease in photocatalytic efficiency. Both ∆_(absorbance)_, which is the difference between the initial absorbance value and the absorbance value at the end of the photocatalytic test, and the apparent rate constant (k_app_) remained nearly constant over the four cycles. This indicates that sample B is not only an efficient photocatalyst but also exhibits stability over multiple cycles. This property is advantageous for practical applications, as immobilized photocatalysts, such as thin films, are more convenient to recycle and are therefore well-suited for long-term use compared to mobilized photocatalysts in powder form.

## 4. Conclusions

In this research, rough nanocomposite TiO_2_/SiO_2_-np films were successfully synthesized in a single step using a combination of reactive sputtering and an aerosol jet. The SiO_2_-np was selected for its inert nature under light allowing the assessment of the morphological and microstructural changes of the matrix induced by the nanoparticle introduction on the photocatalytic activity. This study shows that the incorporation of SiO_2_-np at any stage of the film growth results in the creation of growth defects that lead to the development of the surface, with larger defects observed (≈500 nm) when SiO_2_-np is introduced earlier in the process. Additionally, the incorporation of SiO_2_-np into the TiO_2_ films also slightly restricts the crystal growth of the matrix films. The TiO_2_ crystal quality was decreased (dislocation density, micro-strain, and disorder levels increased). Considering the measurement error, the incorporation of SiO_2_-np did not significantly change the optical bandgap values of the films. In the specific case of TiO_2_/SiO_2_-np, it is demonstrated that the introduction of the nanoparticles only at the beginning of the film growth is more efficient than a continuous introduction, resulting in almost 30% improvement of the photocatalytic activity without further optimization. The continuous introduction of the nanoparticles disturbs the development of large growth defects and consequently limits the surface area. Furthermore, the presence on the surface of non-photoactive particles also reduced the surface area of the photoactive phase. The study also highlights several factors influencing photocatalytic activity, emphasizing the significance of the surface area of the photoactive phase. It is then a preliminary step for the synthesis of more complex nanocomposite films with active nanoparticles. This original process allows the use of a relevant strategy for the nanoparticle introduction according to the required functionality (sensor, hydrophilicity, magnetism, tribology, antireflection, etc.).

## Figures and Tables

**Table 1:** Pulsed DC reactive magnetron sputtering conditions for TiO_2_ matrix thin films synthesis.

Power per Target (W)	Frequency (kHz)	“Off” Time (µs)	Air Flow Rate (sccm)	O_2_ Flow Rate (sccm)	Total Pressure (Pa)	Deposition Rate (nm/min)	Substrate Temperature (°C)
1000	50	4.0	120	40	0.5	11.3	300

**Table 2:** The micro-strain and dislocation density values of the presented samples.

Sample	Micro-Strain (*σ*) (×10^−2^) (nm)	Dislocation Density (δ) (×10^−3^) (lines/nm^2^)
A	20 ± 1	4 ± 1
B	23 ± 1	6 ± 1
C	23 ± 1	6 ± 1
D	26 ± 1	8 ± 1
E	35 ± 1	14 ± 1
F	25 ± 1	7 ± 1

**Table 3:** The bandgap value and Urbach energy value of the TiO_2_/SiO_2_-np nanocomposite films.

Samples	Bandgap Energy (eV)	Urbach Energy (meV)
A	3.26 ± 0.02	137 ± 5
B	3.21 ± 0.02	141 ± 5
C	3.25 ± 0.02	140 ± 5
D	3.27 ± 0.02	214 ± 5
E	3.27 ± 0.02	214 ± 5
F	3.30 ± 0.02	118 ± 5

## Data Availability

The datasets used and/or analyzed during the current study available from the corresponding author on reasonable request.

## Supplementary Materials

The following supporting information can be downloaded at: https://www.mdpi.com/article/10.3390/ma17061227/s1.
