# Supplementary material for: One-Step Synthesis of TiO2/SiO2-np Nanocomposite Photocatalytic Multilayer Films: Effect of Incorporation Time Sequences of SiO2 Nanoparticles during the TiO2 Film Growth
Source: Materials (Basel). 2024 Mar 7;17(6):1227. doi: 10.3390/ma17061227 (PMC10972100; doi:10.3390/ma17061227)
Supplement: Supplementary file 1 [file materials-17-01227-s001.zip › materials-2884820-supplementary.pdf]

materials

MDPI

Supplementary materials

Figure S1 shows a detailed cross-sectional view of the aerodynamic lens, labeled with thin plate diaphragms of decreasing diameter and a trajectory of the nanoparticle flow through these diaphragms, which is increasingly concentrated on the central axis and forms a divergent nanoparticle jet at the exit of the aerodynamic lens.

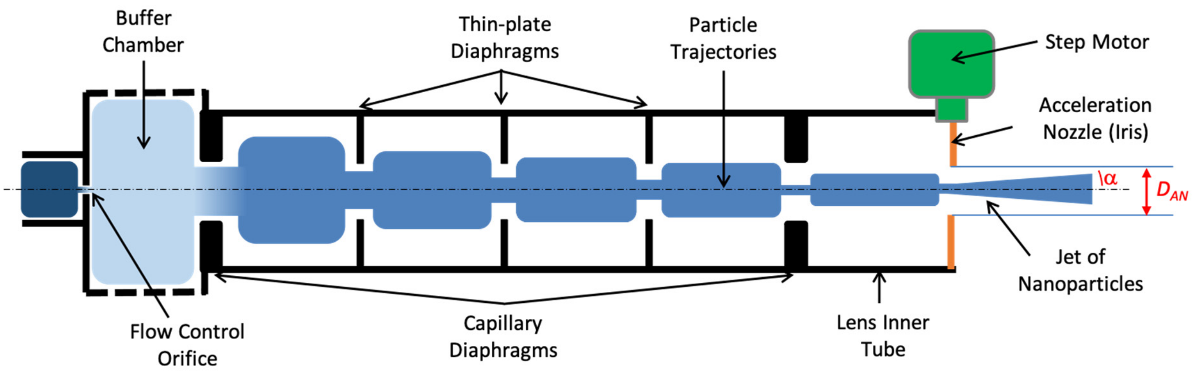

Figure S1. Scheme of a modified aerodynamic lens for the divergent nanoparticle jet formation.

Figure S2 shows as-deposited sample photos on microscopic slide to demonstrate the synthesis of nanocomposite films using P25 nanoparticles as an example. P25 nanoparticles are used for their whiteness, making easier the observation of the nanoparticles.

Figure S2(1) shows the P25 nanoparticle jet formed at the exit of the aerodynamic lens, which appears as a roughly homogeneous white circular P25 nanoparticle deposit. A brighter white spot can be observed in the center of the deposit, corresponding to the inhomogeneous part of this deposit. In order to improve the homogeneity of the deposit, a hexagonal mask was designed to be placed at the exit of the aerodynamic lens (Figure. S2(2)). The mask-treated P25 nanoparticle jet and the sputtered TiO_2 film were then combined to synthesize the TiO_2/P25-np nanocomposite film with a lateral moving of the substate holder. As shown in Figure S2(3), the synthetized white nanocomposite film can basically cover the entire surface of a microscopic slide (25 x 75 mm^2) with an uniform thickness.

To better understand the spatial homogeneity of the nanoparticle jet. The variation in transmittance over the entire spatial distribution of an only Au-np deposit on glass substrate is measured by laser extinction at 532 nm (figure S3). The nanoparticle jet is relatively homogeneous, apart from the fact that a drop in transmittance of over 10 % is observed at the center of the deposit, synonymous with a greater quantity of nanoparticles at the center of the jet. This overintensity of the nanoparticle jet at its center could be due to a partial excess of nanoparticle agglomerates.

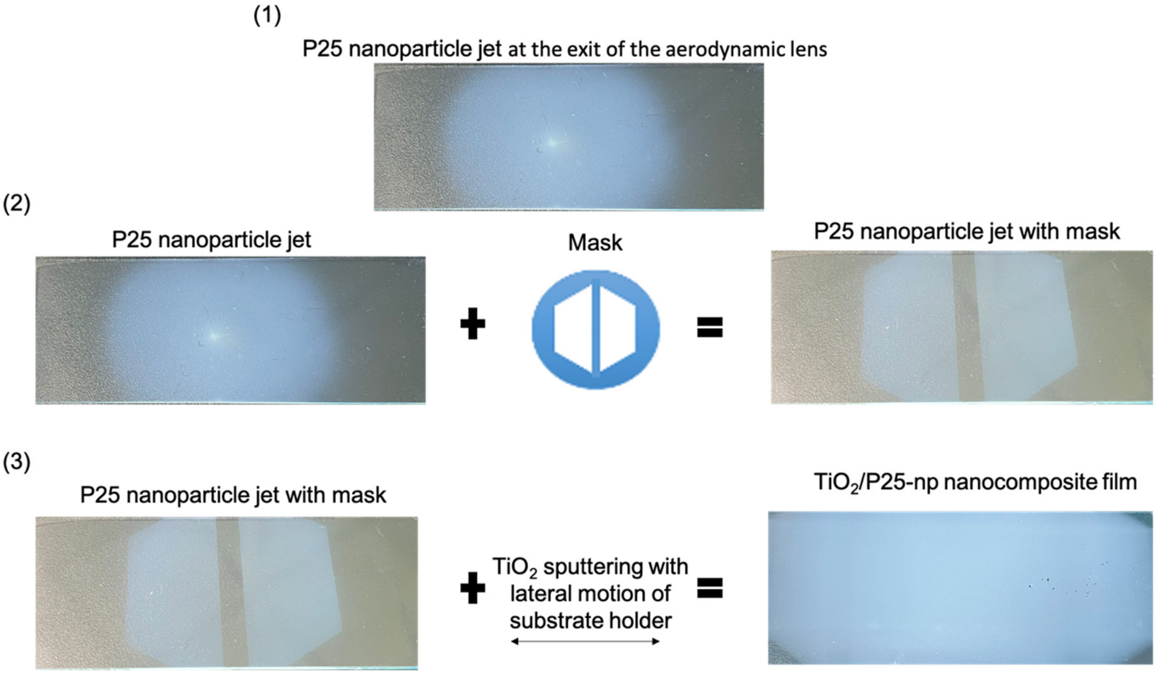

Figure S2. (1) Photo microscopic slide covered with P25 nanoparticle jet at the exit of the aerodynamic lens; (2) photo of microscopic slide covered with P25 nanoparticle jet across a mask placed in front of the lens outlet; (3) photo of microscopic slide covered with a TiO_2/P25-np nanocomposite film obtained with the lateral motion of the substrate holder.

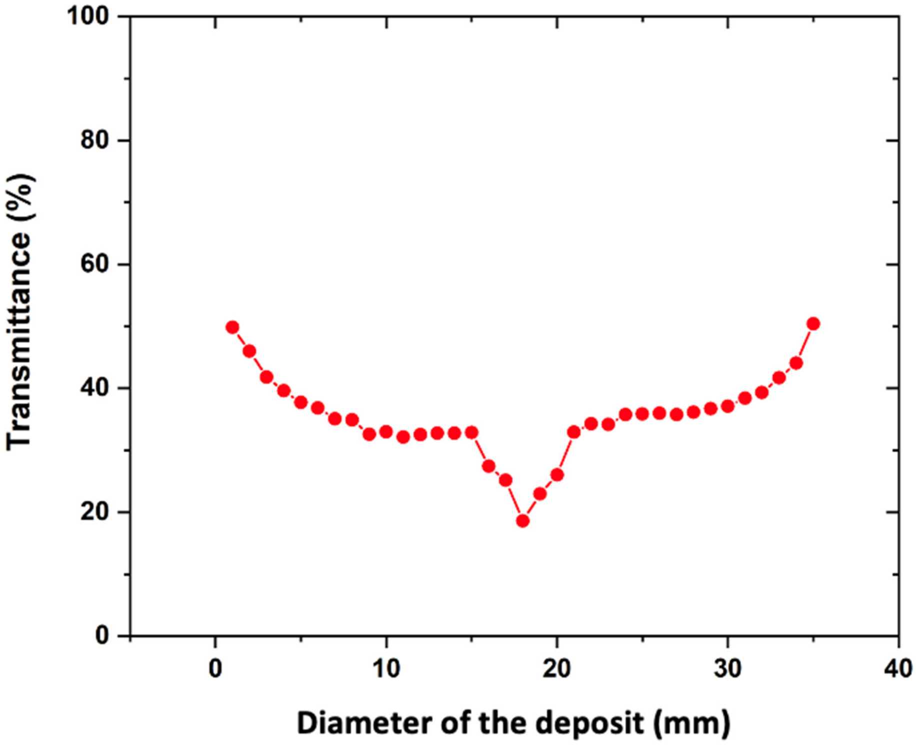

Figure S3. Spatial homogeneity of Au-np deposit without mask (derived from 532 nm laser extinction measurements of Au-np deposit on glass substrates).
